# Mitochondria, Bioenergetics and Excitotoxicity: New Therapeutic Targets in Perinatal Brain Injury

**DOI:** 10.3389/fncel.2017.00199

**Published:** 2017-07-12

**Authors:** Bryan Leaw, Syam Nair, Rebecca Lim, Claire Thornton, Carina Mallard, Henrik Hagberg

**Affiliations:** ^1^The Ritchie Centre, Hudson Institute of Medical Research Clayton, VIC, Australia; ^2^Perinatal Center, Institute of Physiology and Neuroscience, Sahlgrenska Academy, University of Gothenburg Gothenburg, Sweden; ^3^Department of Obstetrics and Gynaecology, Monash University Clayton Clayton, VIC, Australia; ^4^Centre for the Developing Brain, Division of Imaging Sciences and Biomedical Engineering, King’s College London, King’s Health Partners, St. Thomas’ Hospital London, United Kingdom; ^5^Perinatal Center, Department of Clinical Sciences, Sahlgrenska Academy, Gothenburg University Gothenburg, Sweden

**Keywords:** perinatal brain injury, hypoxia-ischemia, mitochondria, neuroprotection

## Abstract

Injury to the fragile immature brain is implicated in the manifestation of long-term neurological disorders, including childhood disability such as cerebral palsy, learning disability and behavioral disorders. Advancements in perinatal practice and improved care mean the majority of infants suffering from perinatal brain injury will survive, with many subtle clinical symptoms going undiagnosed until later in life. Hypoxic-ischemia is the dominant cause of perinatal brain injury, and constitutes a significant socioeconomic burden to both developed and developing countries. Therapeutic hypothermia is the sole validated clinical intervention to perinatal asphyxia; however it is not always neuroprotective and its utility is limited to developed countries. There is an urgent need to better understand the molecular pathways underlying hypoxic-ischemic injury to identify new therapeutic targets in such a small but critical therapeutic window. Mitochondria are highly implicated following ischemic injury due to their roles as the powerhouse and main energy generators of the cell, as well as cell death processes. While the link between impaired mitochondrial bioenergetics and secondary energy failure following loss of high-energy phosphates is well established after hypoxia-ischemia (HI), there is emerging evidence that the roles of mitochondria in disease extend far beyond this. Indeed, mitochondrial turnover, including processes such as mitochondrial biogenesis, fusion, fission and mitophagy, affect recovery of neurons after injury and mitochondria are involved in the regulation of the innate immune response to inflammation. This review article will explore these mitochondrial pathways, and finally will summarize past and current efforts in targeting these pathways after hypoxic-ischemic injury, as a means of identifying new avenues for clinical intervention.

## Introduction

Perinatal brain injury remains a significant cause of long-term neurological and physical disability, and a significant socioeconomic burden in both developed and developing countries (Kruse et al., 2009; Lawn et al., 2011). While preterm birth carries a significantly increased risk of brain injury, babies born term are also at risk of exposure to insults including hypoxic-ischemeic encephalopathy (HIE), metabolic disease, or neonatal stroke (Hagberg et al., 2015). Moderate hypothermia is a validated and increasingly used intervention in infants that develop brain injury, including from hypoxia-ischemia (HI; Hutchison et al., 2008; Azzopardi et al., 2013). However, its availability is typically restricted to developed countries due to the cost of equipment needed as well as the need for a stable supply of electricity to maintain a therapeutic level of cooling (Kumar et al., 2009). While there is recognition that cooling may still be effective in low resource settings that do not have tertiary intensive care, there remains the ethical dilemma of performing clinical trials on neonatal encephalopathy with the addition of normothermia groups (Montaldo et al., 2015; Tagin et al., 2015). Furthermore, hypothermia does not always confer neuroprotection (Azzopardi et al., 2009). There is a need to better understand the underlying pathogenesis of perinatal brain injury to be able to identify new therapeutic targets, and thus reduce the prevalence of neurological impairment and associated lifelong physical disability such as cerebral palsy.

Mitochondria are the powerhouses of the cell, primarily responsible for the production of adenosine triphosphate (ATP) as well as playing regulatory roles in cell death, including autophagy and apoptosis (Nunnari and Suomalainen, 2012). Advances in genomic and proteomic sequencing have provided irrefutable evidence that these vestiges of bacterial ancestry also perform more diverse roles, particularly in disease (Hagberg et al., 2014). These range from early work showing that mutations in mitochondrial DNA (mtDNA) are implicated in diseases such as Parkinson’s disease (Swerdlow et al., 1996), to research on the epigenetic modulation of mtDNA, including methylation by DNA methyltransferases, which add yet another layer in which mitochondria can influence and contribute to disease (van der Wijst and Rots, 2015). There is thus a growing appreciation that identifying and targeting mitochondrial pathways thought to be responsible for the manifestation of initial injury post asphyxia as well as long-term neurodevelopment holds great promise in the field of perinatal medicine. In addition, recent work suggests that mitochondria, and reactive oxygen species (ROS) derived from mitochondria, have important roles in the regulation of inflammation both in response to sterile and microbial insults (Suliman and Piantadosi, 2016). This review article seeks to summarize past therapeutic interventions, as well as current efforts to target these mitochondrial pathways during the fragile early period of development, and to identify new avenues for therapeutic intervention. First, however it is important to understand the different phases of hypoxic-ischemic injury to identify targets for intervention.

## The Phases of Hypoxic-Ischemic Injury

The understanding of the pathogenesis of perinatal brain injury has evolved rapidly in recent years. Initial thoughts centered on a cascade of energy and nutrient deficiency, inflammation, oxidative stress and subsequent cell death and neuronal loss (Volpe, 2001; Hagberg et al., 2015). Currently it is well accepted that HI triggers an acute series of events, as well as a tertiary phase of injury response, which extends days to months after the initial insult (Fleiss and Gressens, 2012). Briefly, the events preceding these later outcomes can be divided into three distinct periods—the primary, secondary and tertiary phases, each identified by different molecular processes as well as the involvement of different cellular subtypes (Fleiss and Gressens, 2012). During the primary phase in the minutes-to-hours following asphyxia, neural energy failure occurs through a rapid decrease of high energy phosphates (phosphocreatine (pCr), ATP). This is followed by a transient recovery to baseline levels during reperfusion generating a “latent” phase therapeutic window. A secondary “delayed” phase subsequently occurs from hours to days post injury characterized by an apoptotic-necroptotic cell death phenotype (Northington et al., 2011). The tertiary phase is thought to be responsible for persisting effects years after injury, including immune memory (Hoeijmakers et al., 2016) and proper development of white matter tracts in adulthood (Favrais et al., 2011). This suggests that inflammation during the early perinatal phase can modify later risk to developing neurological and psychiatric disorders (Hagberg et al., 2012). Mitochondria play essential roles in both proper neurodevelopment as well as in response to HI injury pathogenesis (Hagberg et al., 2014), so effective targeting of mitochondria derangement might have therapeutic merit, especially during this open therapeutic window.

## Physiological Roles of Mitochondria, Structure, Trafficking

Mitochondria are small membrane-enclosed organelles, remarkably mobile and plastic, associated with ATP generation, calcium regulation and the biosynthesis of amino acids, lipids and nucleotides (Green et al., 2011).

**Figure 1:**
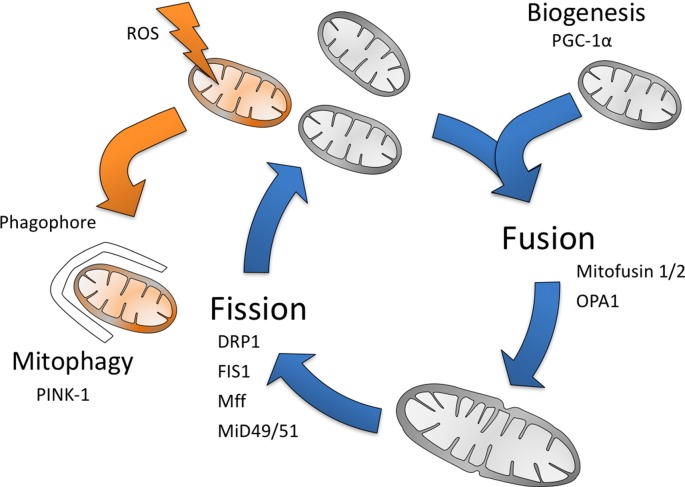
The mitochondrial life cycle. Mitochondria turnover occurs continuously throughout life, with mitochondria constantly being degraded (mitophagy) and replaced (biogenesis). Mitochondria are also able to produce reactive oxygen species (ROS), which causes mitochondrial membrane destabilization and damage to their proteins and DNA, resulting in their degradation. Mitochondria also undergo fragmentation (fission) and fusion, which are vital to many cellular processes and in particular allow adaptation to cellular needs (Gottlieb and Carreira, 2010).

Mitochondria constantly change their shape and undergo fusion, fission, migration biogenesis and degradation (mitophagy); matching the cellular needs (Archer, 2013; Figure 1). In order to understand variations in mitochondria function and consequent selective vulnerability to injury, the organelle must be placed within the context of its cellular, functional, developmental and neuroanatomical environment (Dubinsky, 2009; Rintoul and Reynolds, 2010). The location of mitochondria in the cell varies between cell types, but they are most often localized near sites of high ATP utilization as their major role is to produce and supply energy, ATP, to the cells through the enzyme complexes forming the respiratory chain. Electron flow through the electron transport chain (ETC) generates a proton gradient across the inner mitochondrial membrane, which drives the production of ATP by ATP synthase. Under normal conditions, this machinery provides almost all (>90%) of the ATP in the brain (Hagberg et al., 2014). However, a small proportion of electrons escape the ETC complexes I, II and III under normal conditions to react with oxygen to form superoxide (${\text{O}}_{2}^{-}•$; Grivennikova et al., 2010). Mitochondrial function is critically important during brain development and throughout life in metabolic tasks and for the regulation of cell death.

## Mitochondrial Morphobiogenesis: Physiological Mechanisms and Brain Development

### Mitophagy

The mitochondrial genome is unprotected and mtDNA repair is inefficient compared with that in the nucleus (Hiona et al., 2010). Damaged mitochondria can generate large amounts of ROS which is not only toxic to mtDNA but can promote lipid peroxidation, mitochondrial dysfunction, impaired cellular function and induce apoptosis (Murphy, 2009; Grivennikova et al., 2010). Therefore, damaged and dysfunctional mitochondria are constantly being degraded (mitophagy) to reduce the oxidative load and are replaced by new mitochondria through mitochondrial biogenesis (Gottlieb and Carreira, 2010). This process is particularly important in long-lived non-proliferative cells such as neurons. Dissipation of mitochondrial membrane potential results in accumulation of the kinase PINK-1 at the outer mitochondrial membrane which phosphorylates ubiquitin and parkin resulting in binding of mitophagy receptors (e.g., optineurin, NDP52) to the mitochondrial surface. The latter will recruit the autophagy machinery proteins (e.g., ULK1, DFCP1 and WIPI1) and the mitochondria will be wrapped into the autophagosome and degraded by fusion with lysosomes (Lazarou et al., 2015; Pickrell and Youle, 2015). The importance of mitophagy for brain development remains unknown. However, deletion of the autophagy (Atg5 or Atg7) genes results in neurodegeneration during early life in mice (Hara et al., 2006; Komatsu et al., 2006) and suppression of genes involved in lysosomal function causes severe central nervous system (CNS) abnormalities (Levine and Kroemer, 2008). More specifically to mitophagy, mutations in the human genes Parkin and PINK-1 result in a juvenile form of neurodegeneration (Levine and Kroemer, 2008).

### Biogenesis

Mitochondrial biogenesis usually occurs starting from already existing mitochondria and needs proteins encoded by both nuclear and mtDNA (Scarpulla, 2011). Peroxisome proliferator-activated receptor gamma coactivator 1-alpha (PGC-1α) is the key regulator in this process, forming complexes with DNA and supporting the function of transcription factors such as nuclear respiratory factors (Wu et al., 1999; Huss et al., 2002). These factors regulate transcription of nuclear genes encoding mitochondrial proteins. Additionally, the mitochondrial transcription factor A (TFAM) is expressed from nuclear DNA, translated in the cytosol, and transported into the mitochondria (Attardi and Schatz, 1988). TFAM drives the transcription of 13 additional key enzymes needed for assembly of the ETC, encoded within the mitochondrial genome, together with the RNA needed for their translation (Larsson et al., 1998). During neuronal differentiation, the number of mitochondria per cell increases, and inhibition of mitochondrial biogenesis impairs neuronal differentiation (Vayssière et al., 1992; Mattson et al., 2008). PGC-1α is expressed at high concentrations in neurons due to their high energy demands (Andersson and Scarpulla, 2001).

Overexpression of PGC-1α in cultured neurons increases the number of dendritic spines and promotes synaptic differentiation, whereas deletion of the gene for PGC-1α, has the opposite effect (Cheng et al., 2012). In addition, mice with knockdown of the PGC-1α gene exhibit progressive neuropathology and abnormal behavior (Lin et al., 2004; Cheng et al., 2012). These data strongly suggest that mitochondrial biogenesis has an important regulatory role in synaptic and brain development.

### Fusion/Fission

Mitochondria constantly fuse and divide which appears to be crucial for a number of functions such as the maintenance of organelle function, mediating DNA or protein quality control and repair of injured mitochondria (Tanaka and Youle, 2008). The process is driven by dynamin-related protein 1 (DRP1) recruited to mitochondria by fission proteins (e.g., FIS1, Mff, MiD49/51; Sheridan and Martin, 2010; van der Bliek et al., 2013). Mitofusins 1 and 2 mediate fusion of the outer mitochondrial membrane, whereas Optic atrophy 1 (OPA1) fuses the inner membrane (Benard and Karbowski, 2009). The fusion–fission cycle is critical for embryonic and brain development (Benard and Karbowski, 2009). Mitochondrial fission appears essential for dendritic development. Disruption of DRP1-dependent fission leads to very elongated mitochondria in Purkinje cells resulting in abnormal spines, dendrites and synapses, and ultimately in ataxic behavior (Liu and Shio, 2008). Overexpression of dominant-negative alleles of the gene that encodes DRP1, leads to fewer spines and synapses, reversed by DRP1 which increases the density of dendritic spines (Li et al., 2004). Cultured neurons deficient in DRP1 have decreased numbers of neurites and defective synapses (Ishihara et al., 2009); a dominant-negative mutation of the DRP1 gene was found in a newborn girl with microcephaly and abnormal brain development (Waterham et al., 2007). These results support that fission is an important means to increase the number of mitochondria to meet energy demands during neuronal plasticity. Inability to meet these demands has severe effects on CNS development. Mitochondrial fusion is also critical for brain development. Mitofusin 1 and mitofusin 2 are both essential for embryonic CNS development in mice and for cerebellar development. A mutation in mitofusin 2 causes the neurodegenerative disorder Charcot-Marie-Tooth neuropathy type 2A (Chen et al., 2003; Zuchner et al., 2004). Additionally, overexpression of OPA1 leads to a decrease in dendritic spines, and OPA1 gene mutation causes autosomal-dominant optic atrophy type 1 (Delettre et al., 2000; Li et al., 2004). Mitochondrial fusion proteins may also attenuate apoptosis by inhibiting the release of proapoptotic agents like cytochrome c (cyt c), while mitochondrial fission protein DRP1 promotes apoptosis through Bax, leading to mitochondrial outer membrane permeabilization and cell death (Cassidy-Stone et al., 2008).

## Mitochondrial Role in Apoptosis and Secondary Brain Injury

Apoptosis (programmed cell death) is essential for the normal development of tissues and is especially key in neuronal development (Raff et al., 1993). The balance between cell survival and cell death is therefore required to be highly regulated; as such it is unsurprising that aberrant activation of apoptotic pathways occurs in several neurological conditions including stroke and a variety of neurodegenerative diseases (Vila and Przedborski, 2003). Cellular apoptosis can be achieved through two routes, the extrinsic pathway activated in response to extracellular signals such as the cell death receptor Fas and tumor necrosis factor alpha (TNF-α) and mediated by death receptors (Green, 2000) and the intrinsic pathway activated in response to DNA damage or cellular stress. Although each pathway has unique members, both mechanisms may converge downstream at the level of the mitochondrion, where if the insult is severe enough, there is catastrophic permeabilization from which the cell cannot recover. It is important to note that mechanisms of mitochondrial permeabilization are age-dependant and while Cyclophilin D is critical in the adult brain, Bax-dependent mechanisms dominate in the immature brain (Wang et al., 2009).

Mitochondrial permeabilization results in the release of mitochondrial apoptogenic factors into the cytosol including apoptosis-inducing factor (AIF), endonuclease g (Endo G), cyt c and Smac/Diablo. These proteins have a number of pro-apoptotic functions; cyt c interacts with Apaf-1 to form an active apoptosome, providing a platform for procaspase-9 cleavage; Smac/Diablo interacts with inhibitors of apoptosis (IAP) reducing their negative influence on the activity of caspases (Vila and Przedborski, 2003). In contrast with cyt c and Smac/Diablo, AIF and Endo G operate through a caspase-independent pathway. Both are translocated to the nucleus from the mitochondria in response to death—inducing stimuli where they induce fragmentation of nuclear DNA (Susin et al., 1999; Li et al., 2001).

Some plasma membrane receptors contain the so-called death domain in their intracellular domain (e.g., TNF-R1, DR4, DR5, Fas) and are able to trigger apoptosis when activated from the binding of the corresponding ligand (e.g., TNF-α, TRAIL, FasL). This extrinsic pathway of apoptosis continues with the activation of a death-inducing signaling complex (DISC) adjacent to the death domain of the receptor. Activated DISC catalyzes the proteolytic cleavage and transactivation of procaspase-8 (Love, 2003). Activated caspase-8 either directly activates caspase-3 or mediates cleavage of Bcl-2 interacting domain (BID) to truncated Bid (tBid), which integrates different death pathways at the mitochondria (Sugawara et al., 2004). tBid translocates to the mitochondria where it interacts with other proapoptotic proteins and triggers the release of apoptogenic factors like cyt c and AIF from the mitochondria. Apoptosis then proceeds in the same way as for the intrinsic pathway with caspase-dependent and caspase-independent cell death regulated by mitochondria. There is ample evidence to suggest that both the intrinsic (Zhu et al., 2000, 2005) and to some extent the extrinsic pathway (Graham et al., 2004; Kichev et al., 2014) are critical contributors to immature brain injury (see this issue). In addition, AIF is released from mitochondria after neonatal HI (Zhu et al., 2003) and binds to cyclophilin A in the cytosol and the complex translocates to the nucleus and induces DNA damage (Zhu et al., 2007a) and contributes to brain injury (Zhu et al., 2007b). Notably, the caspase-dependent route appears to be more important in females while the AIF pathway is more predominant in males (Zhu et al., 2006; Johnston and Hagberg, 2007). The knowledge about mitochondrial physiology and its role in cell death has expanded dramatically over the past decade. Nonetheless, more detailed information regarding the role of mitochondria in perinatal injury and brain development is urgently needed in order to develop more effective mitoprotective therapies.

## Contribution of Mitochondria to Inflammation

Inflammation is an important risk factor for injury in the developing brain (Strunk et al., 2014; Hagberg et al., 2015; Lai et al., 2017). Mitochondria regulate innate immune responses and play a direct role in the assembly of innate sensing machineries that trigger the host immune response. This is achieved mainly through transcriptional regulation of inflammatory cytokines/chemokines and their maturation by inflammasomes (Monlun et al., 2017). Mitochondria converge on signaling pathways involved in inflammation through: (a) mitochondrial ROS (mtROS) production; (b) mtDNA release; and (c) mitochondrial antiviral signaling protein (MAVS). These act as key triggers in the activation of innate immune response following a variety of stress signals that include infection, tissue damage and metabolic dysregulation (Sandhir et al., 2017). ROS produced by mitochondria, are a major host defense mechanism since they act as a crucial signaling molecule and as mediators of inflammation.

The movement of ${\text{O}}_{2}^{-}•$ across mitochondrial membranes is highly limited because of its negative charge. However, the presence of transmembrane proteins, such as voltage-dependent anion channels (VDAC) found in mitochondria, allow trans-membrane passage of ${\text{O}}_{2}^{-}•$ produced in ETC (Han et al., 2003). Thereby allowing access to cytosolic targets leading to multiple functional outcomes such as activation of redox-sensitive transcription factors like hypoxia inducible factor 1 alpha (HIF-1α) and NF-κB, causing activation of pro-inflammatory cytokines and inflammasomes (Chandel et al., 2000; Wang et al., 2010).

Pathogen-associated molecular patterns (PAMPs) and damage-associated molecular patterns (DAMPs) bind to specific receptors including RIG-I-like receptors (RLRs), NOD-like receptors (NLRs) and Toll-like receptors (TLR), to generate cytokines that are essential for eliminating pathogens or repairing tissue damage (Mogensen, 2009). mtDNA is a rich source of DAMPs which activate several innate immune pathways involving toll-like receptor 9 (TLR9), NLRP3 and STING signaling thereby resulting in effector responses (Weinberg et al., 2015). Systemic activation of toll-like receptor 2 (TLR2) induces brain inflammation and increases the vulnerability to HI, possibly through suppression of mitochondrial respiration (Mottahedin et al., 2017a,b).

During viral infection, the pattern recognition receptors RIG-I and MDA5 attach to viral RNA interacting the mitochondrial polypeptide adaptor, MAVS, which drives the production of type I interferon (Saitoh and Akira, 2010). Studies have demonstrated that viral-mediated disruption of mtDNA homeostasis serves as a cell-intrinsic indicator of infection that works in parallel with virus sensing mechanisms to engage antiviral innate immunity (West et al., 2015). Interestingly, recent studies have demonstrated that mtDNA induces lung and liver inflammation (Zhang et al., 2010). It is also proposed that mitochondrial DAMPs drive hyperactivation of innate immunity, which might underlie systemic inflammatory response syndrome (Tait and Green, 2012).

### Effect of Inflammation on Mitochondrial Metabolism

As the site of cellular respiration and energy production, mitochondrial metabolism is one of the central processes affected by inflammation. Acutely triggered immune responses, as well as chronic inflammation, are characterized by significant changes in mitochondrial metabolism. This can result in shifts in energy supply/demand causing metabolic acidosis and hypoxia, thereby triggering phenotypic shifts in immune cells like microglia. Thus, strategies directed at controlling excessive inflammation mediated by mitochondria through metabolic control may represent novel preventive and therapeutic interventions.

Mitochondria can exert immune regulation at different levels by manipulation of metabolic pathways, thereby allowing an appropriate cytokine response to each situation, which is crucial for the correct establishment of immune responses (Monlun et al., 2017; Tur et al., 2017). In macrophages, activation via the lipopolysaccharide (LPS)/Toll-like receptor 4 (TLR4) pathway cause accumulation of Krebs cycle intermediates such as succinate in the mitochondria, resulting in the stabilization of HIF-1α and promoting inflammatory gene expression, such as induction of interleukin-1 beta (IL-1β; Tannahill et al., 2013; Mills et al., 2016).

The switch from oxidative phosphorylation (OXPHOS) to glycolysis, a phenomenon similar to the Warburg effect, is an important concept for understanding metabolic changes occurring in immune cells upon activation (Kelly and O’Neill, 2015). The preferential use of glycolysis over OXPHOS, although inefficient in terms of total energy production, allows immune cells to churn-out ATP and intermediates for cytokine production at a faster rate (Marelli-Berg et al., 2012; Chang et al., 2013). In anti-microbial defense mechanisms, such as neutrophil extracellular traps (NETs), neutrophils derive energy from glycolysis as they contain very few mitochondria. NET formation is inhibited by the glycolysis inhibitor, 2-deoxy-glucose, and to a lesser extent by the ATP synthase inhibitor oligomycin (Rodríguez-Espinosa et al., 2015). These examples provide evidence of cross talk between metabolism and immune response and thus the possibility of metabolism-based immune regulation.

Mitochondrial metabolism is necessary for T cell activation and pharmacologic inhibition of OXPHOS and glycolysis *in vitro* ablate T cell proliferation (Chang et al., 2013). Activated T cells isolated from a mouse model of systemic lupus erythematosus (SLE) are dependent on mitochondrial metabolism (Wahl et al., 2010) and furthermore, peripheral blood lymphocytes from patients with SLE have increased mitochondrial metabolism and ROS production (Gergely et al., 2002).

Release of mtDNA can cause activation of inflammasomes resulting in caspase-1-dependent secretion of the inflammatory cytokines IL-1β and IL-18, and an inflammatory form of cell death referred to as pyroptosis (Yu et al., 2014). Pyroptosis has been defined as a type of programmed cell death triggered by pathological stimuli and is important in controlling microbial infections (Bergsbaken et al., 2009). NLRP3-mediated inflammatory signaling, IL-1β production and pyroptosis in macrophages causes a disruption of glycolytic flux, an important signal for host-cell response to the intracellular pathogen, which disrupt metabolism by uptake of host-cell glucose (Sanman et al., 2016).

Recent data indicate that TFAM when released from necrotic cells could act as a specific DAMP causing a pro-inflammatory and cytotoxic responses (Little et al., 2014). TFAM exacerbates the inflammatory response in T cells through lysosomal dysfunction (Baixauli et al., 2015). Following necrosis, TFAM acts as a danger signal enhancing the plasmacytoid dendritic cell (pDC) response by binding to the receptor for advanced glycation end products (RAGE) and TLR9 (Cherry et al., 2016).

**Figure 2:**
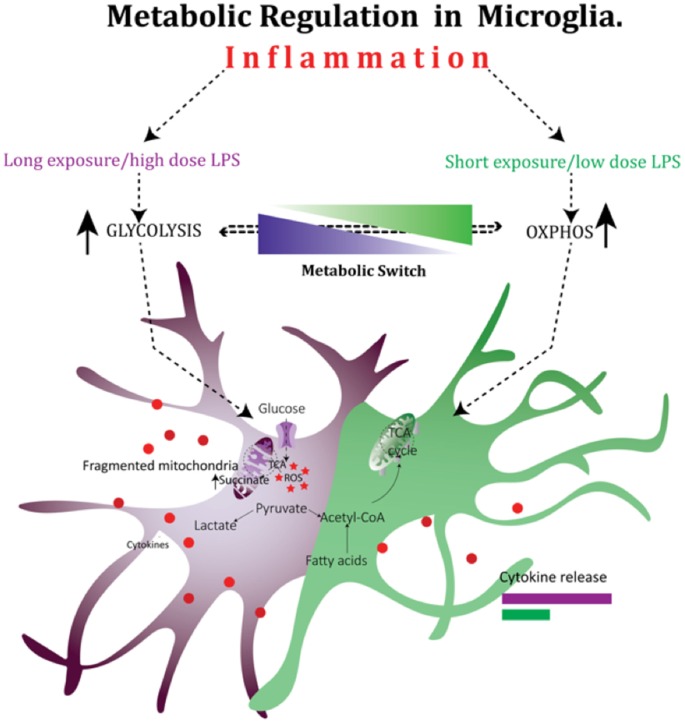
Mitochondria play a critical role in the regulation of cellular metabolism in microglia after exposure to lipopolysaccharide (LPS). Metabolic reprogramming in microglia cells during inflammation: in microglia cells, a short exposure to a low dose of LPS causes a transient increase in oxidative phosphorylation (OXPHOS). This increase in OXPHOS is however suppressed in response to a prolonged exposure to high dose of LPS, forcing a shift in metabolism towards glycolysis to match the metabolic demand.

It is clear from above that there is a link between mitochondrial function, inflammation and energy metabolism in immune cells, however, such effects have thus far remained poorly characterized in the brain. In microglia cells, a short exposure to a low dose of LPS causes a transient increase in OXPHOS (Figure 2). This increase in OXPHOS is however suppressed in response to a prolonged exposure to high dose of LPS, forcing a shift in metabolism towards glycolysis to match the metabolic demand. This LPS-induced metabolic reprogramming is directly related to mitochondrial dynamics as preventing excessive mitochondrial fission in microglia by a DRP-1 inhibitor reverses the LPS induced metabolic switch and reduces the pro-inflammatory cytokine output in microglia (Nair et al., 2016). Thus, in addition to their well-appreciated roles in cellular metabolism and programmed cell death, mitochondria appear to have a cardinal role in regulating the immune system. In summary, patients with inflammatory diseases may benefit from detailed evaluation of mitochondrial function and suitable metabolic support to improve immune dysfunction. Altering mitochondrial dynamics may be a therapeutic modality for preventing neuroinflammation-induced microglia over-activation and may prevent neuronal cell death associated with various neurodegenerative processes. This is of particular importance given the lack of treatment options for perinatal brain injury despite many showing promise at the pre-clinical phases.

## Therapies for Perinatal Brain Injury

Despite improvements in the standard of care, the only “treatment” available for perinatal brain injury is preventative in nature—clinical hypothermia. Hypothermia, or cooling of the infants’ body temperature to 33–34°C within several hours of an hypoxic-ischemic event, was first shown in a pilot study to reduce both mortality and long term motor outcomes (Eicher et al., 2005), and is now widely established in neonatal care (Shankaran et al., 2005; Azzopardi et al., 2009; Edwards et al., 2010). Despite this, hypothermia is not always effective, e.g., with head cooling studies showing no effect in infants with severe brain injury after HIE (Gluckman et al., 2005). A factor contributing to the variance in therapeutic efficacy is the requirement of maintaining core body temperature within a small and narrow window, which is particularly challenging in low resource settings (Kumar et al., 2009). To overcome these caveats, hypothermia has been used in combination with other experimental therapies that have shown promise in clinical trials.

## Combination Treatments Approved for Clinical Trials

### Erythropoietin (EPO)

Erythropoietin (EPO) is a naturally produced angiogenic hormone having neuroprotective, neurogenic (Wang et al., 2004) as well as anti-inflammatory (Sun Y. et al., 2005; Rees et al., 2010) and anti-apoptotic (Kellert et al., 2007) properties. Recombinant human EPO (rhEPO) is safe for administration and is currently used to treat patients with low circulating levels as a result of chronic kidney disease or chemotherapy. Importantly, EPO is an activator of mitochondrial biogenesis (Carraway et al., 2010), and within the CNS appears to work by increasing the expression of the mitochondrial regulator PGC-1α in astrocytes and neurons (Horng et al., 2015). In a rodent model of adult stroke, rhEPO also increased vascular endothelial growth factor (VEGF) and brain-derived neurotrophic factor (BDNF), stimulating recovery as well as reducing stroke infarct size and improving functional outcomes (Wang et al., 2004). rhEPO also ameliorated hyperoxia-induced apoptosis and inflammation in neonatal mice (Kaindl et al., 2008; Sifringer et al., 2010), possibly via modulation of key autophagic proteins (Bendix et al., 2012).

In humans, an early clinical trial showed infants with HIE treated with a combination of rhEPO and hypothermia, had improved neurological outcomes, as well as fewer white matter tract abnormalities (Elmahdy et al., 2010). Even though EPO is one of the most promising neuroprotective interventions currently undergoing clinical testing, a recent 2-year follow-up study in very preterm infants treated with EPO (NCT00413946) showed no demonstrable differences in neurodevelopmental outcomes (Natalucci et al., 2016). However, in a Chinese study preterm infants were randomly assigned to receive rhEPO (500 IU/kg; *n* = 366) or placebo (*n* = 377) intravenously within 72 h after birth and then once every other day for 2 weeks (Song et al., 2016). The rate of moderate/severe neurological disability in the rhEPO group (7.1%) was significantly lower compared to the placebo group (18.8%; *p* < 0.001; Song et al., 2016). Furthermore, the considerable variation in between timing and dosage even in pre-clinical studies (see commentary by Juul, 2013) can mean the difference between improved neurological outcomes (Fan et al., 2013) and no therapeutic merit (Fang et al., 2013).

### Melatonin

Melatonin, more commonly known as the sleep hormone, has been widely used as an anti-oxidative therapy in experimental models for a number of years (Balduini et al., 2012; Manchester et al., 2015). Melatonin works primarily via scavenging free radicals and stimulating the innate anti-oxidant system (Barlow-Walden et al., 1995). There is evidence that melatonin pre-treatment is neuroprotective in a rat model of middle cerebral artery occlusion (MCAO; Pei and Cheung, 2004), and furthermore, this protection was observed when melatonin was administered to the fetus directly (Welin et al., 2007) or via the maternal circulation (Miller et al., 2005). After umbilical cord occlusion, melatonin significantly reduced DNA and RNA fragmentation, apoptosis, as well as inflammation and astroglial activation (Miller et al., 2005). Crucially, melatonin can also promote white matter maturation, one of the key deficits in diseases of childhood disability such as cerebral palsy (Olivier et al., 2009). Recently mitochondria have been postulated as a major synthesis site for melatonin (He et al., 2016), suggesting that mitochondria may play a critical role in its anti-oxidative and therapeutic properties. Indeed, melatonin administration has been shown to be mitoprotective via acting as a mitochondrial antioxidant (Martin et al., 2000) and can also directly inhibit the opening of the mitochondrial permeability transition pore (mtPTP), responsible for release of calcium and cyt c from the mitochondria (Andrabi et al., 2004). Melatonin also modulates mitochondrial bioenergetics, decreasing mitochondrial fission and increasing mitochondrial fusion after oxidative stress (Parameyong et al., 2015; Chuang et al., 2016), and regulating autophagy and mitophagy (see review by Coto-Montes et al., 2012). In clinical trials, melatonin combined with clinical hypothermia improved white matter tract development and reduced apoptosis in a piglet model of HIE (Robertson et al., 2013) and a phase I clinical trial is currently underway (NCT02621944).

### Stem Cell Therapies

There has been a focus on cell therapies in the field of regenerative medicine in recent years (Baraniak and McDevitt, 2010). Cell therapies have been heralded as the next pillar of modern medicine due to their reported multiple modes of action, touted as an advantage over traditional pharmacological agents that typically only target a single pathway in the pathophysiology of perinatal brain injury. Umbilical cord blood cells (UCBs) are one of the most well-studied and characterized cells in perinatal brain injury to date. UCBs consist of a combination of cell types including hematopoietic stem cells, endothelial progenitors, lymphocytes, monocytes and mesenchymal stem cells (MSCs; Pimentel-Coelho et al., 2012). UCBs have anti-inflammatory and anti-apoptotic properties in animal models of HIE, with their primary mode of action being the modulation of immune cells after injury (Schwarting et al., 2008), protecting from hypoxia-induced apoptosis (Hall et al., 2009) and release of trophic factors that promote neural recovery and repair post-ischemia such as BDNF and neurotrophins (Fan et al., 2005; Newman et al., 2006). During injury these UCBs, including MSCs, hone towards sites of injury, their migratory ability powered via interactions with chemokine and cytokine receptors including the fractalkine receptor CX_3_CR1 (Ji et al., 2004). Once there, MSCs drive neuronal differentiation after injury, promote cellular regeneration (Busch et al., 2011) and confer anti-inflammatory properties which reduce immune cell proliferation and activation (Li et al., 2005), and improve long-term motor outcomes (Van Velthoven et al., 2010). Recent evidence however suggests that MSCs can also participate in transfer of mitochondria via structures termed tunneling nanotubes (TNTs; Hsu et al., 2016). TNT formation is a result of F-actin polymerization, and is driven by activation of the pro-apoptotic p53 signaling within the stressed cell and downstream activation of the Akt/PI3K/mTor pathways (Wang et al., 2011). Co-culture of MSCs and human umbilical vein endothelial cells (HUVECs) exposed to glucose-oxygen deprivation resulted in the formation of TNTs and transfer of mitochondria from MSCs to HUVECs, restoring functional aerobic respiration and reducing apoptosis (Liu et al., 2014). This transfer has also been observed in corneal epithelium (Jiang et al., 2016) or pulmonary epithelium providing protection (Wecht and Rojas, 2016).

Tissue banking following birth is a growing market, and while UCB administration has been proven safe (Sun W. et al., 2005), there is some concern that few patients ever require therapy, with some approximating that 1 in 3000 children will require transfusion (Nietfeld et al., 2008). Together UCBs have been utilized in 30 clinical trials worldwide as an intervention for cerebral palsy (Clinicaltrials.gov, search query “umbilical cord cells” AND “cerebral palsy”), with one study completed (NCT01193660, Clinicaltrials.gov). The study showed that a combination therapy of UCBs and EPO resulted in significant improvements in motor and cognition, with associated improvements in structural and metabolic changes in the brain (Min et al., 2013).

## Mitochondrial-Targeted Therapies

Whilst it is clear that broadly targeting excitotoxicity has some merit, it is clear from the mixed success of ion channel blockers that more targeted therapies are required in perinatal brain injury. Mitochondria play a pivotal role in bioenergetics, cell-cycle regulation and the oxidative stress response, and thus represent an emerging target for neuroprotective therapies.

### Protecting from Mitochondrial Permeabilization

#### Mitochondrial Calcium Uniporter (Mcu)

Mitochondrial calcium uniporter (Mcu) is the pore-forming complex located on the mitochondrial inner membrane, and is responsible for fine-tuning the mitochondrial membrane potential (Oxenoid et al., 2016). Mcu plays a crucial role in mediating NMDA-receptor-induced excitotoxic death (Stout et al., 1998), as it allows Ca^2+^ influx into the mitochondria, perpetuating the downward apoptotic spiral. Crucially, knockdown of Mcu stabilizes the mitochondrial membrane and confers resistance to excitotoxicity, as well as neuroprotection as modulated by synaptic firing by adjacent neurons (Qiu et al., 2013; Utkina-Sosunova et al., 2013). This opens up the possibility that targeting of Mcu by pharmacological agents might have therapeutic merit in clinical conditions (Camara et al., 2010, 2011) where excitotoxicity-induced cell death is a major pathological feature.

#### Sigma-1 Receptors

Other ways of preventing mitochondrial destabilization are via modulators such as the sigma-1 receptor, located on the endoplasmic reticulum. The administration of 4-Phenyl-1-(4-phenylbutyl)piperidine (PPBP), a sigma-1 agonist, reduced neuronal death *in vitro* and in an *in vivo* model of excitotoxic developmental brain injury, with a concomitant decrease in microglial activation and loss of mitochondrial membrane potential (Wegleiter et al., 2014). Other sigma-1 agonists such as dehydroepiandrosterone (DHEA; Hashimoto et al., 2006) and allopregnanolone (Shirayama et al., 2011) have also been shown to improve cognitive deficits and infer anti-depressant effects in models of neuropsychiatric disorders, likely via interactions with NMDA receptors.

#### Mitochondrial ATP-Sensitive K^+^ (mitoK_ATP_) Channel Openers

The mitochondrial ATP-sensitive K^+^ (mitoK_ATP_) channel is essential for the tightly regulated leak of K^+^ ions across the mitochondrial membrane, allowing precise control over its membrane potential (Facundo et al., 2006). Interestingly, opening mitoK_ATP_ channels by compounds such as diazoxide result in protection from ischemic damage as well as reducing mtROS release (Facundo et al., 2007) and cell death (Fornazari et al., 2008). In a piglet model of HIE, diazoxide protects the integrity of the mitochondrial membrane and accumulating Ca^2+^ levels in CA1 pyramidal neurons (Domoki et al., 2004), and in a mouse model of MCAO confers neuroprotection to hypoxic neurons via modulation of pro- and anti-apoptotic proteins along the Bax-Bcl2 pathways (Liu et al., 2002). Furthermore, opening of the mitoK_ATP_ channel seems to be critical for induction of tolerance in the brain, a phenomenon whereby a sub-threshold insult of e.g., hypoxia, ischemia or a drug renders the CNS resistant to a second severe insult (Sanders et al., 2010; Hagberg et al., 2014). However, there is some contention as to whether diazoxide works entirely via mitoK_ATP_ channels, as the use of diazoxide in submitochondrial particles from pig heart did not affect both mitochondrial membrane potential and nicotinamide adenine dinucleotide (reduced form) (NADH) oxidation (Hanley et al., 2002). Further research have since corroborated these findings (Anastacio et al., 2013; Coetzee, 2013) and identified numerous other pathways by which diazoxide exerts its physiological effects, including activating cardiovascular and endothelial K_ATP_ channels (Coetzee, 2013) and regulating the release of neurotransmitters such as norepinephrine (Mohan and Paterson, 2000) and acetylcholine (Kilbinger et al., 2002).

### Directly Targeting Mitochondrial Downstream Apoptotic Pathways

#### Caspase Inhibitors

Caspases such as caspase-3 and -8 play a pivotal role in the early stages of apoptosis after HI injury. This is particularly true for perinatal brain injury (Hu et al., 2000), as altered caspase activity during this crucial period interrupts the programmed cell death critical for the proper functional development of the CNS (Raff et al., 1993). Early efforts using specific and pan-caspase inhibitors had mixed success, with some studies showing reduced neuronal apoptosis (Cheng et al., 1998) and necrosis (Han et al., 2002) after HIE; however another study reported no benefit of pre-treatment with a pan-caspase inhibitor (Joly et al., 2004). The lack of protection may be due to differences in the bioavailability of the pharmacological compounds, or more likely reflects the importance of caspase-independent death pathways in perinatal brain injury. More recent efforts have turned to the generation of safer, more specific and pharmacokinetically enhanced inhibitors. Both quinoline-Val-Asp(Ome)-CH2-O-phenoxy (Q-VD-OPh) and the methyloxyphenylketone (mOPh) derivative, TRP601, were effective in neonatal models of ischemic injury, reducing infarct size, improving neurological function (Renolleau et al., 2007) and attenuating glial activation and inflammation (Chauvier et al., 2011). TRP601 preferentially inhibits caspase-2, an initiator caspase positioned upstream of mitochondrial permeabilization. TRP601 targets caspase-2 activation preventing truncation of Bid, and translocation of Bax to the mitochondrial membrane for mitochondrial permeabilization and release of pro-apoptotic proteins such as cyt C (Carlsson et al., 2011; Chauvier et al., 2011).

#### Phosphatase and Tensin Homolog Deleted on Chromosome 10 (PTEN) Modulators

Phosphatase and tensin homolog deleted on chromosome 10 (PTEN) is a negative regulator of members of the PI3K/Akt signaling pathway, responsible for regulating cell growth and survival. PTEN is also thought to couple PI3K/Akt signaling to the pro-death c-Jun N-terminal kinase (JNK) pathway. As PTEN has phosphatase activity, the usage of specific tyrosine phosphatase inhibitors such as bpv(pic) have been used to rescue neuronal cell death after ischemic injury via blocking PTEN-mediated downregulation of the PI3K pathway and decoupling from JNK1/2 signaling (Zhang et al., 2007). In a mouse model of kainate-induced excitotoxicity, bpv(pic) also displayed anti-inflammatory properties, reducing reactive astrogliosis and mitochondria apoptosis (Grande et al., 2014). PTEN inhibition has also been shown to rescue cortical neurons after HI in the immature brain (Zhao et al., 2013) and neuroprotective trophic factors such as IGF-1, growth hormone and hexarelin (Gustafson et al., 1999; Brywe et al., 2005a,b) all seem to act by enhancing PI3K/Akt signaling preventing BAX dependent mitochondrial permeabilization.

#### JNK Inhibitors

Activation of the JNK pathways leads to further downstream pro-apoptotic pathways including BCL-2 (Jin et al., 2006) and MAPK-activating death domain-containing protein (MADD; Centeno et al., 2007). Interestingly, Jnk3^−/−^ mice are resistant to ischemic (Kuan et al., 2003), hypoxic-ischemic (Pirianov et al., 2007) and excitotoxic brain injury (Brecht et al., 2005), thus raising the possibility of targeting JNK activity as a therapy for perinatal brain injury. The JNK inhibitor SP600125 reduced infarct size and reduced apoptosis primarily via blockade of mitochondrial translocation of pro-apoptotic proteins Bax and Bim, and thus release of cyt c (Gao et al., 2005). Interestingly from a therapeutic standpoint, blockade of JNK activity was neuroprotective when administered either before or after ischemia (Guan et al., 2006) and via either intracerebral or intravenous injection (Guan et al., 2006). JNK inhibitors also have long-lasting activity, improving white matter development as well as cognitive and motor function in rats with HIE up to 14 weeks after injury (Nijboer et al., 2010, 2013).

#### p53 Inhibitors

p53, along with Ca^2+^ leak and ROS generation, are the major causes of mitochondrial permeabilization via interactions with the pro-apoptotic BCL-2 family (Galluzzi et al., 2009). Reduction of available p53 also reduces binding to DNA sites responsible for cell death (Leker et al., 2004) as well as reduced expression of BAX and caspase activity (Culmsee et al., 2001). Blocking p53 association with the mitochondrial membrane with pifithrin-μ reduced cerebral damage, ROS production and improved sensorimotor function after 6–10 weeks post-HI (Nijboer et al., 2011). There is some debate however whether the primary mode of action of pifithrin-μ is via p53 blockade as its use accompanied with partial or complete neuronal p53 deletion confer no or limited neuroprotection (Baburamani et al., 2017).

It is evident that directly targeting mitochondrial pathways in pre-clinical studies have shown a plethora of therapeutic merit. However studies have also reported that indirect protection of mitochondria via maintaining the homeostasis of the CNS microenvironment, or blocking subsequent energy failure, could also have merit.

### Indirect Protection of Mitochondrial Function

#### Creatine

Creatine is a vital component in both aerobic respiration and ATP recycling in all tissues of the body. Creatine is used to enhance athletic performance by a biphasic “loading” period involving ingestion of 20 g/day for the first week and then a “maintenance” phase of 2 g/day (Hultman et al., 1996), resulting in sustained improvements in high-intensity exercise performance and lean body mass (Buford et al., 2007). In traumatic brain injury, creatine improved mitochondrial bioenergetics and reduced ROS production (Sullivan et al., 2000), and reduced the severity of cerebral infarcts in a model of unilateral carotid artery ligation (Berger et al., 2004). In perinatal brain injury, creatine supplementation reduces ATP depletion after ischemic injury (Brewer and Wallimann, 2000) thereby protecting neurons from oxygen-glucose deprivation-mediated apoptosis and necrosis (Balestrino et al., 2002). In models of ischemia, creatine supplementation has been shown to reduce severity of infarcts and improving neurological function (Lensman et al., 2006) likely via improving cerebral blood flow (Prass et al., 2007). There is also limited evidence that creatine has anti-oxidant properties, scavenging hydroxyl free radicals and affording cytoprotection (Sestili et al., 2011). From a clinical standpoint, while creatine weakly crosses the blood-brain barrier (BBB), creatine can also be supplemented maternally as creatine can cross the placental barrier, and supplementation via this route is also neuroprotective in a spiny mouse model of birth asphyxia (Ireland et al., 2011). Efforts to create more lipophilic Cr-derived compounds resulted in the generation of PCr-Mg-complex acetate (PCr-Mg-CPLX), which readily crossed the BBB (Lunardi et al., 2006) and when administered in a model of MCAO improved stroke and behavioral outcomes (Perasso et al., 2009).

#### Dichloroacetate (DCA)

Dichloroacetate (DCA) is a small molecule inhibitor of pyruvate dehydrogenase (PDH) kinase (PDHK), which regulates the activity of PDH. PDH is responsible for the conversion of pyruvate to acetyl-CoA, a critical component of the Krebs cycle. DCA is used in clinic to treat congenital lactic acidosis (Berendzen et al., 2006), of which 90% of reported cases are a result of a genetic defect in the α subunit of PDH (Lissens et al., 2000). Importantly, long-term administration of DCA is well-tolerated in children (Stacpoole et al., 2008) and is able to cross the BBB (Williams et al., 2001). DCA was initially used in models of ischemic-reperfusion injury as a means of reducing secondary energy failure, with administration of DCA increasing levels of ATP and PCr 4 h after ischemic injury (Katayama and Welsh, 1989) and also decreasing levels of lactate within the brain (Kaplan et al., 1987). Recently, intraperitoneal administration of DCA in a model of unilateral HI reduced infarct size and apoptosis via increased mitochondrial biogenesis as a result of increased availability of mitochondrial acetyl-CoA (Sun et al., 2016). More studies are required to assess DCA as a potential therapy after HI injury.

#### Protecting Astrocytes

Astrocytes are the most abundant cell-type in the brain, crucial for maintenance of optimal bioenergetics in the brain as well as providing trophic support and neurotransmitter release. High densities of mitochondria have been localized in the fine astrocytic processes responsible for regulating cerebrovascular flow (Zonta et al., 2003), and therefore neurovascular coupling (Otsu et al., 2015). However, these processes are damaged after ischemia (Ito et al., 2009), leading to neuronal dysfunction and death (Bambrick et al., 2004). Protecting astrocytes could have therapeutic merit, given their ability to re-uptake glutamate, delaying or preventing excitotoxic injury. Furthermore, astrocytic mitochondrial function is restored between 30 min and 1 h after ischemic injury (Morken et al., 2014), substantially quicker than neuronal mitochondrial recovery, and as such are ideally placed to reduce the impact in the secondary phase of hypoxia-induced injury (Berger et al., 2016). *In vivo*, astrocytes participate in mitochondrial transfer towards injured neurons, improving cell survival and neurological outcomes, a mechanism which is governed by CD38 signaling (Hayakawa et al., 2016). Modulation of astrocytic signaling, such as inhibition of the SNAP (Soluble NSF Attachment Protein) REceptor (SNARE) pathway also influences NMDA receptor expression and therefore sensitivity towards excitotoxic injury (Hines and Haydon, 2013). Administration of anti-oxidants such as resveratrol, melatonin or nicotinamide adenine dinucleotide phosphate (reduced form) (NADPH) inhibitors are protective towards astrocytic function after ischemia (Fernández-Gajardo et al., 2014), via inhibition of apoptotic pathways and stabilization of mitochondrial function (Lin et al., 2014).

## Conclusion

The only clinically approved therapy for a hypoxic-ischemic event, whole-body hypothermia, has been shown to be effective at neuroprotection and reducing mortality rates. However, no therapies exist that target mitochondria and the stages of perinatal brain injury once they have manifested, critically the latent phases are responsible for later neurological impairment and disabilities such as cerebral palsy.

Evidence in recent years has identified mitochondrial dysfunction and downstream activation of pro-apoptotic pathways as a major target for future therapies. In pre-clinical models, these therapies have shown promise at not just reducing inflammation but also improving neurological outcomes weeks after the onset of ischemic injury. Critically, therapies targeting multiple pathogenic pathways, including cell therapies, are currently in late phase clinical trials.

Advances in technology may soon allow researchers to overcome the major problem of CNS therapies, namely the ability to cross the BBB. Nanoparticle-based drug delivery systems including synthetic polyamidoamine dendrimers have been used to great effect in an animal model of cerebral palsy, showing localization to glial cells and suppressing neuroinflammation (Kannan et al., 2012). Compounds such as creatine can also be infused in large amounts into albumin-coated gold nanospheres, providing a quicker and more effective administration route to cross the BBB (López-Viota et al., 2009). However, more research into the exact roles that mitochondria play in disease and subsequent therapy is needed, with a particular emphasis on more accurately mapping the chronological events downstream of perinatal brain injury and thus identifying therapeutic windows for novel intervention.

## Author Contributions

All authors contributed to the writing of the manuscript as well as critical revision. All authors read and approved the final manuscript.

## Conflict of Interest Statement

The authors declare that the research was conducted in the absence of any commercial or financial relationships that could be construed as a potential conflict of interest.

## Abbreviations

AIF: apoptosis-inducing factor
ATP: adenosine triphosphate
BBB: blood-brain barrier
BDNF: brain-derived neurotrophic factor
BID: Bcl-2 interacting domain
CNS: central nervous system
Cyt c: cytochrome c
DAMPs: damage-associated molecular patterns
DCA: dichloroacetate
DHEA: dehydroepiandrosterone
DISC: death-inducing signaling complex
DRP1: dynamin-related protein 1
Endo G: endonuclease G
EPO: erythropoietin
ETC: electron transport chain
HI: hypoxia-ischemeia
HIE: hypoxia-ischemeic encephalopathy
HIF-1α: hypoxia inducible factor 1 alpha
HUVECs: human umbilical vein endothelial cells
IAP: inhibitors of apoptosis
IL-: interleukin
JNK: c-Jun N-terminal kinase
MADD: MAPK-activating death domain-containing protein
MAVS: mitochondrial antiviral signaling protein
MCAO: middle cerebral artery occlusion
Mcu: mitochondrial calcium uniporter
mitoKATP: mitochondrial ATP-sensitive K+
mOPH: methyloxyphenylketone
MSCs: mesenchymal stem cells
mtDNA: mitochondrial DNA
mtPTP: mitochondrial permeability transition pore
mtROS: mitochondrial ROS
NAD: nicotinamide adenine dinucleotide
NADH: nicotinamide adenine dinucleotide (reduced form)
NADPH: nicotinamide adenine dinucleotide phosphate (reduced form)
NETs: neutrophil extracellular traps
NLRs: NOD-like receptors
${\text{O}}_{2}^{-}•$: superoxide
OPA1: optic atrophy 1
OXPHOS: oxidative phosphorylation
pCR: phosphocreatine
pDC: plasmacytoid dendritic cell
PDH: pyruvate dehydrogenase
PGC-1α: peroxisome proliferator-activated receptor gamma coactivator 1-alpha
PPBP: 4-Phenyl-1-(4-phenylbutyl)piperidine
PTEN: phosphatase and tensin homolog deleted on chromosome 10
Q-VD-OPh: quinoline-Val-Asp(Ome)-CH2-O-phenoxy
RAGE: receptor for advanced glycation end products
rhEPO: recombinant human EPO
RLRs: RIG-I-like receptors
ROS: reactive oxygen species
SLE: systemic lupus erythematosus
tBid: truncated Bid
TFAM: mitochondrial transcription factor A
TLR-: Toll-like receptor
TNF-α: tumor necrosis factor alpha
TNTs: tunneling nanotubes
UCBs: umbilical cord blood cells
VDAC: voltage-dependent anion channels
VEGF: vascular endothelial growth factor.
